# Incorporation of Solid Lipid Nanoparticles into Stirred Yogurt: Effects in Physicochemical and Rheological Properties during Shelf-Life

**DOI:** 10.3390/nano13010093

**Published:** 2022-12-25

**Authors:** Raquel F. S. Gonçalves, Rui Rodrigues, António A. Vicente, Ana C. Pinheiro

**Affiliations:** 1 CEB—Center of Biological Engineering, University of Minho, 4710-057 Braga, Portugal; 2 LABBELS—Associate Laboratory, Braga/Guimarães, Portugal

**Keywords:** lipid-based nanodelivery systems, curcumin, nanostructures in food, food fortification, functional foods, storage period

## Abstract

The aim of this work was to develop a yogurt fortified with curcumin. Curcumin is a lipophilic compound with a wide range of biological activities; however, it presents low water solubility and low bioavailability, and therefore it was the first to be encapsulated in solid lipid nanoparticles (SLNs). Then the influence of the incorporation of curcumin-loaded SLNs on the physicochemical (i.e., pH, titratable acidity, syneresis and color) and rheological properties of yogurt during its shelf-life (30 days at 4 °C) was evaluated. SLN incorporation into yogurt did not affect pH and titratable acidity compared to the control (i.e., plain yogurt) during shelf-life, even though the yogurt with SLNs presented lower values of pH (4.25 and 4.34) and acidity (0.74% lactic acid and 0.84% lactic acid) than the control in the end, respectively. Furthermore, the yogurt with SLNs presented slightly higher values of syneresis than the control during the shelf-life; however, it did not present visual differences in whey separation. Relative to the color, the incorporation of SLNs into the yogurt imparted a strong yellow color to the sample but did not affect color stability during shelf-life. Both samples showed flow curves with yield stress and shear-thinning behavior during shelf-life, and, regarding the viscoelastic behavior, both showed a typical weak viscoelastic gel with an elastic structure. Overall, curcumin-loaded SLNs incorporation did not affect the physicochemical and rheological stability of yogurt during shelf-life, showing a promising application for the development of new functional foods.

## 1. Introduction

Currently, due to the increasing concern of consumers for a healthier lifestyle, one of the main objectives of food researchers and producers is to develop and manufacture functional foods with improved nutritional and health properties. Functional foods are defined as natural or processed foods that when consumed regularly within a varied diet, can have positive health effects beyond basic nutrition, such as reduction of the risks of non-communicable diseases. Processed functional foods can be fortified with nutraceuticals (e.g., vitamins, minerals, phenolics, essential fatty acids, etc.) or one of their undesirable ingredients (e.g., salt, fat, sugar, preservatives, flavors or colors) can be removed or replaced with sustainable and natural alternatives [1,2].

Yogurt is a fermented milk product produced by *Streptococcus thermophilus* and *Lactobacillus delbrueckii* spp. *Bulgaricus*. Yogurt is more nutritious than milk and an optimal source of protein, phosphorus, calcium, magnesium, zinc, riboflavin, thiamin, vitamin B12 and niacin [3]. Furthermore, it can be produced in several styles (e.g., stirred, kefir, Greek, drinkable, among others), flavors (e.g., strawberry, banana, vanilla, etc.) and fat content (i.e., full, low or non-fat) [4]. When yogurt becomes highly consumed globally and is used as a nutritional component’s delivery vehicle in the human diet, the fortification of this product (e.g., with curcumin) is a good strategy for enhancing the nutrient intake provided by daily food products [3]. Curcumin is a lipophilic nutraceutical that can be found in *Curcuma longa*. Even though curcumin has already been used in the food industry as a colorant as well as a spice, it has only gained interest from food scientists due to its health-promoting benefits, such as anti-inflammatory, antioxidant, antimicrobial and anticarcinogenic activities, over the past few decades. However, curcumin has low solubility and quick degradation in aqueous solutions, a high rate of metabolic degradation and low bioavailability [5]. Additionally, the incorporation of nutraceuticals (such as curcumin in its free form) into food products can present several limitations, such as the chemical degradation of nutraceuticals by the exposure of the food to several operational and environmental conditions, the impact (often deleterious) on the organoleptic properties of the food product, and the possible molecular interactions between the nutraceutical and food ingredients changing the nutraceutical’s bioactivity [2]. Therefore, nanoencapsulation can be one of the strategies to overcome these limitations while improving the food products’ fortification.

Solid lipid nanoparticles (SLNs) are one of the lipid-based nanostructures in which curcumin can be encapsulated due to its lipophilic character. SLNs can be produced using one or more solid lipids, such as waxes, fatty acids, glycerides and triacylglycerol, forming fully crystallized droplets at room temperature where the nutraceutical is accommodated [6]. The solid lipid matrix of SLNs can enable the protection of the nutraceutical from high thermal conditions, pH and high ionic strength during its processing, storage and intake [7].

The incorporation of nanoparticles into food products can present some challenges, due to the physical and chemical interactions present in the food matrix that can impact the food’s properties, particle’s stability, nutraceutical’s bioaccessibility and bioactivity [8]. Although it is crucial to understand the possible effects caused by the nanostructures when incorporated within food products during their processing and storage, there are still only a few works that study these effects using bio-based nanoparticles [9,10,11,12], but none using SLNs. In a previous work from our group, SLNs, nanoemulsions (NE) and nanostructured lipid carriers (NLC) were evaluated in terms of curcumin’s bioavailability, particle behavior during in vitro digestion, cellular permeability and toxicity after in vitro digestion. The results showed that despite the SLNs presenting the lowest curcumin bioaccessibility when compared to NE and NLC, this nanostructure presented good particle stability in the gastrointestinal tract and no toxicity effects after in vitro digestion, conversely to NE and NLC [13]. Furthermore, in another work from our group, SLNs, NLC and NE were subjected to different food simulants, where they were evaluated in terms of curcumin’s in vitro release kinetics and particle stability and were incorporated within a model beverage. Their physicochemical properties were evaluated during the storing period and curcumin’s bioaccessibility after in vitro digestion was assessed. SLNs exhibited good particle stability and lower curcumin release as well as NLC, when subjected to acetic acid 3%, which simulates food products with pH below 4.5 (e.g., yogurt). Furthermore, SLNs did not affect the beverage stability during the storing period and presented higher bioaccessibility than NLC and free curcumin [14].

In this work, a curcumin-loaded SLN was incorporated into yogurt in order to develop a yogurt fortified with curcumin, improving curcumin’s stability and bioavailability through encapsulation without interfering with the yogurt’s stability. Therefore, the goal of this work was to evaluate the effect of curcumin-loaded SLNs on the yogurt’s stability during its shelf-life in terms of pH, acidity, color, syneresis and rheological properties.

## 2. Materials and Methods

### 2.1. Materials

Beeswax produced by Apis mellifera was purchased from QUIMIND (Porto, Portugal), PHOSPHOLIPON^®^ 90G, composed by 90% of phosphatidylcholine from soybean, was kindly provided by Lipoid (Steinhausen, Switzerland). Curcumin from Curcuma longa (Turmeric) was purchased from Sigma-Aldrich (St. Louis, MO, USA) and Tween^®^ 80 was obtained from Panreac (Barcelona, Spain). Plain yogurts were purchased at a local supermarket (Braga, Portugal).

### 2.2. SLN’s Production

SLNs were produced as described in Gonçalves et al. (2021) [13]. Briefly, the lipid phase, composed of 3% of beeswax, 1.5% of lecithin (PHOSPHOLIPON^®^ 90G) and 0.1% of curcumin was heated in a water bath at 80 °C under magnetic agitation until full curcumin solubilization. The aqueous phase, constituted by 1.5% Tween^®^ 80 and distilled water, was also heated at 80 °C and solubilized in an Ultra-Turrax homogenizer (T18, Ika-Werke, Germany) for 2 min at 3400 rpm. Then, the aqueous phase was rapidly added to the lipid phase and mixed in an Ultra-Turrax homogenizer at 18,000 rpm for 8 min. The resulting mixture was slowly dispersed at a volume ratio of 1:10 in cold water at 2 ± 1 °C under stirring at 2000 rpm. The SLNs were stirred at 800 rpm for more 35 min to ensure crystallization and stability. The SLNs were freeze-dried using 2% of sucrose as a cryoprotectant.

### 2.3. Particle Size and ζ-Potential

SLNs’ particle size, polydispersity index (PDI), and ζ-potential were measured before and after freeze-drying through dynamic light scattering (DLS) (Zetasizer Nano SZ, Malvern, Worcestershire, UK). All samples were diluted with distilled water. All measurements were performed in triplicate.

### 2.4. Yogurt Preparation

The yogurt fortified with curcumin-loaded SLNs was prepared by adding 6.67 g of SLNs (corresponding to 20 mg of curcumin) into 100 g plain yogurt and mixing manually until obtaining a homogenized mixture. The curcumin amount incorporated into the yogurt respects the acceptable daily intake value (i.e., 3 mg.kg^−1^ body weight/day) [15]. The control yogurt was plain yogurt mixed for the same time as the fortified yogurt. Both the sample and control were stored in glass containers protected from the light at 4 ˚C for 30 days. The sample and the control were prepared in triplicate and the physicochemical and rheological properties were evaluated weekly.

### 2.5. Physicochemical Properties of Yogurt

#### 2.5.1. pH and Titratable Acidity

The pH was measured using a pH meter (HI 2210, HANNA Instruments, Leighton Buzzard, UK). The titratable acidity was determined by mixing the yogurt sample (10 g) with 75 mL of distilled water and titrating the mixture with 0.1 mol·L^−1^ NaOH to pH 8.3 using an auto-titration unit (pH-stat method) (Titrando 902, Metrohm, Herisau, Switzerland). The results were expressed as % of lactic acid [9,10].

#### 2.5.2. Syneresis

The syneresis was analyzed according to de Campo et al. (2019) [9]. Briefly, 20 g of yogurt sample was centrifuged at 3000× *g* for 10 min at 4 °C (Multifuge X3R, Thermo Scientific, Waltham, MA, USA). The supernatant was collected and weighed. The syneresis was calculated according to Equation (1).

(1) $$Syneresis=\frac{Total\;weight\;of\;separated\;liquid}{Total\;weigth\;of\;yogurt}\times 100$$

#### 2.5.3. Color Analysis

The colorimetric parameters (i.e., L*a*b*) were measured using a colorimeter (CR-400, Konica Minolta, Japan) according to the Commission Internationale de l’Eclairage, and the total color difference (Δ*E*) was subsequently calculated as follows (2):

(2) $$\Delta E=\sqrt{\Delta a^{*2}+\Delta b^{*2}+\Delta L^{*2}}$$

#### 2.5.4. Rheological Properties

Rheological measurements were performed in triplicate at 4 ˚C in a TA Instruments HR-1 rheometer equipped with a Peltier plate (TA Instruments, New Castle, DE, USA), using a parallel plate geometry (Ø 40 mm) and a gap of 1 000 μm. The viscoelastic properties of the yogurts were assessed by the frequency sweep determination, between 0.1 and 10 Hz at 0.5% strain. These conditions were within the linear viscoelastic region. The storage modulus (*G′*) and loss modulus (*G″*) were recorded as a function of frequency. Additionally, the flow curves were obtained through 3 steps program (up-down-up) using a continuous ramp and shear rate range from 0.1 to 300 s^−1^. The three steps program was performed in order to eliminate time dependence. The flow behavior was characterized by the Herschel-Buckley model (3) that was fitted to the upward flow curve:

(3) $$\sigma =\sigma_{0}+k\times {\dot{\gamma }}^{\eta }$$

where *σ*_0_ represents yield stress (Pa), *k* consists in the consistency index (Pa.s^n^), $\dot{\gamma }$ is the shear rate (s^−1^) and *η* is the flow behavior index (i.e., *n* = 1 for Newtonian, *n* < 1 for pseudoplastic, and *n* > 1 for dilatant fluids).

### 2.6. Statistical Analyses

The assays were conducted in triplicate and presented as mean ± standard deviation (SD). Statistical analyses were performed using OriginPro 2018 Statistic software (version b9.5.1.195; OriginLab Corporation, Northampton, MA, USA). Data were analyzed using one-way analysis of variance (ANOVA), and the significant differences between the mean values were evaluated using Tukey’s test (*p* < 0.05).

## 3. Results and Discussion

### 3.1. SLN’s Characterization

As presented in our previous work, after production, SLNs showed a particle size of 145.4 ± 8.1 nm, a polydispersity index (PDI) value of 0.253 ± 0.010 and a ζ-potential value of −23.6 ± 1.3 mV [13]. After resuspension, lyophilized SLN showed a slight increase of particle’s size and PDI, 172.4 ± 6.0 nm and 0.282 ± 0.006 respectively, and a slight decrease of ζ-potential, −13.5 ± 0.8 mV. Even though lyophilized SLN maintained stability for one month, it was suitable to be incorporated into the yogurt.

### 3.2. pH and Titratable Acidity

**Figure 1:**
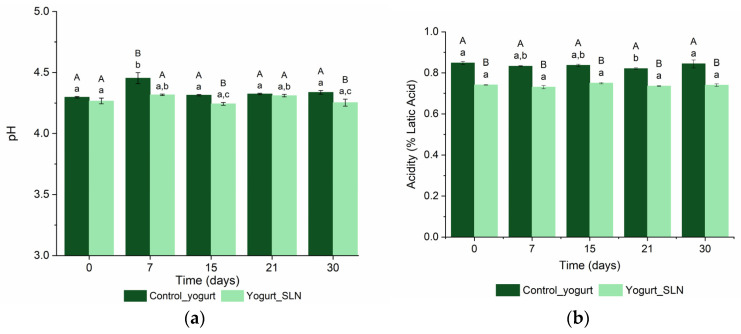
**The** pH (**a**) and titratable acidity (**b**) of control yogurt (i.e., plain yogurt) and yogurt with SLNs incorporated during the shelf-life. ^a–c^ Different lower-case letters indicate a statistically significant difference over time for the same sample (*p* < 0.05). ^A,B^ Different uppercase letters indicate a statistically significant difference between samples for the same time (*p* < 0.05).

The pH and acidity are two important parameters that determine the quality, shelf-life stability and acceptance of yogurt products. Furthermore, pH is also important to access the safety of a food product (pH > 4.5 can allow the survival of some pathogens, such as salmonellae and Escherichia coli) [16]. The pH values and the titratable acidity, determined as the percentage of lactic acid, of control yogurt (i.e., plain yogurt) and yogurt with SLNs during the shelf-life (30 days) at 4 °C are presented in Figure 1a,b, respectively. The pH values ranged between 4.30 ± 0.005 and 4.34 ± 0.015 for control yogurt and 4.27 ± 0.023 and 4.25 ± 0.029 for yogurt with SLNs, during the 30 days of shelf-life, Figure 1a. In a standard plain yogurt, a pH of 4.2 is acceptable to most tastes [16]; therefore, the yogurt with SLNs could be accepted by most consumers in terms of its pH. Overall, the incorporation of SLNs into the yogurt slightly decreased the pH; however, both samples maintained the pH during shelf-life, showing that the SLNs did not impact the stability of the yogurt in terms of its pH. In relation to the titratable acidity, both control yogurt and yogurt with SLNs maintained the titratable acidity over the storage time, where yogurt with SLNs showed lower values than the control yogurt, 0.74% lactic acid and 0.84% lactic acid respectively (*p* < 0.05).. Other authors observed a decrease in pH and an increase in titratable acidity over time for both control yogurts and yogurts fortified with nanostructures due to the production of lactic acid from lactose by the starter culture bacteria [9,12,17]. However, these authors also produced the yogurt in the lab, while in this work a commercial product was used. In conclusion, the incorporation of SLNs into yogurt decreased slightly, but significantly, the pH and titratable acidity values, but these did not change significantly over time.

### 3.3. Syneresis

**Figure 2:**
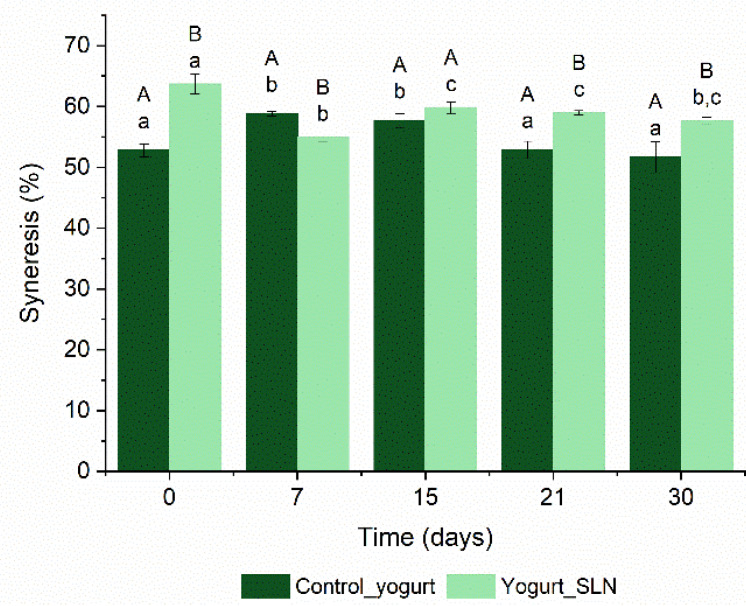
Syneresis values of control yogurt (i.e., plain yogurt) and yogurt with SLNs during 30 days of shelf-life. ^a–c^ Different lower-case letters indicate a statistically significant difference over time for the same sample (*p* < 0.05). ^A,B^ Different uppercase letters indicate a statistically significant difference between samples at the same time (*p* < 0.05).

Syneresis is another important parameter when evaluating the yogurt’s quality and comprises the separation of whey from the gel matrix after its shrinkage [18]. Figure 2 shows the syneresis values of control yogurt and yogurt with SLNs during the shelf-life at 4 °C. The syneresis ranged between 52.8 ± 1.04% and 51.7 ± 2.54% for control yogurt, while for yogurt with SLNs ranged between 63.7 ± 1.62% and 57.7 ± 0.53%, during the 30 days of shelf-life. The incorporation of SLNs into the yogurt slightly increased the syneresis values compared to the control yogurt (*p* < 0.05). This could be explained by the hydrophobic interactions between the nanoparticles and the proteins [19]. The hydrophobic interactions between SLNs and the protein can decrease protein-protein interactions, thereby decreasing the network stability and therefore decreasing water holding capacity within the gel matrix. Furthermore, there was a slight decrease in syneresis after the first week for the yogurt with SLNs (*p* < 0.05), remaining stable until day 30, while the control yogurt maintained the whey separation through the 30 days. This syneresis decrease could be explained by the casein spatial rearrangement within the matrix, resulting in improved protein-protein interactions, and therefore in the decrease of syneresis [19]. Despite the different particle surface properties, these results are in accordance with the work of other authors. For instance, de Campo et al. (2019) reported higher syneresis for the samples with nanoemulsions and nanoparticles compared with plain yogurt and observed a decrease in these values after the first week during 28 days of storage [9]. Zhong et al. (2018) also reported higher values of syneresis for yogurt with nanoemulsions when compared to plain yogurt and a decrease during the 21 days of storage [10].

Despite the slight changes, the samples did not present visual differences (results not shown) on whey separation during shelf-life, showing the visual stability of the samples, which is crucial for the quality and consumer’s acceptance of yogurts.

### 3.4. Color

**Figure 3:**
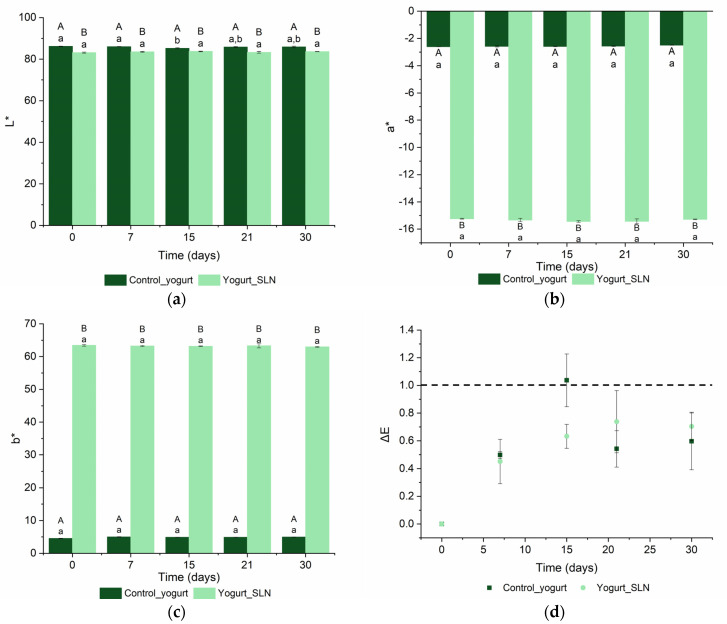
Color parameters: L* (**a**), a* (**b**), b* (**c**) and total color difference (Δ*E*) (**d**) of control yogurt (i.e., plain yogurt) and yogurt with SLNs during shelf-life. ^a–b^ Different lower-case letters indicate a statistically significant difference over time for the same sample (*p* < 0.05). ^A,B^ Different uppercase letters indicate a statistically significant difference between samples for the same time (*p* < 0.05).

**Figure 4:**
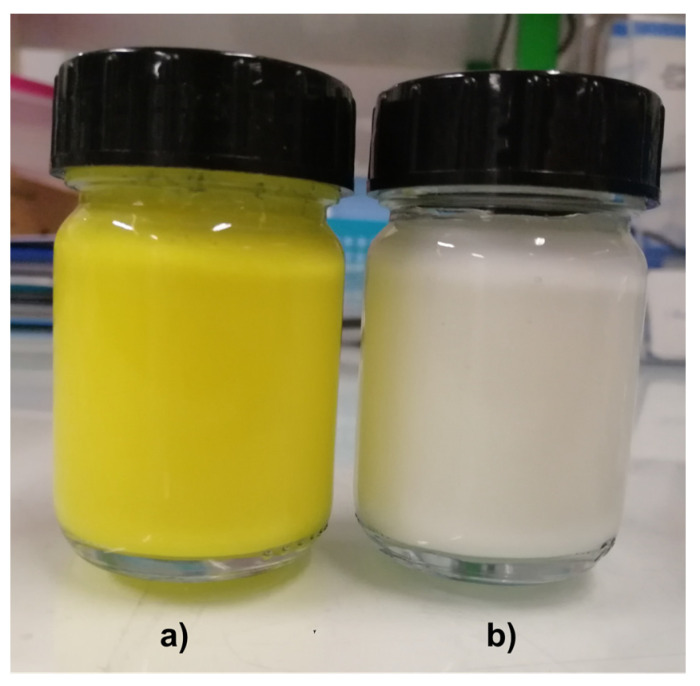
Yogurt with curcumin (**a**) and control yogurt (i.e., plain yogurt) (**b**).

Color is an important parameter for the indication of the deterioration of a product and consumer acceptance. The color changes of yogurt with SLNs and control yogurt over time are shown in Figure 3. In terms of lightness (L*), the values ranged between 86.2 ± 0.07 and 85.8 ± 0.42 for control yogurt and from 83.2 ± 0.28 to 83.6 ± 0.04 for yogurt with SLNs, at 0 and 30 days, Figure 3a. Both samples maintained their values during the shelf-life (*p* > 0.05); however, the yogurt with SLNs showed values slightly lower than the control yogurt (*p* < 0.05). Regarding the parameters a* and b*, the yogurt with SLNs showed much higher values than the control yogurt (*p* < 0.05) Figure 3b,c, which is due to the strong yellow color conferred by curcumin, Figure 4. Moreover, both samples also maintained their values during the shelf-life, showing that SLNs did not release curcumin to the yogurt matrix. On the other hand, other authors incorporated zeaxanthin nanoemulsions and nanoparticles into yogurt that was refrigerated at 4 °C for 28 days and evaluated the color parameters (i.e., L*, a*, b*). They observed some changes of a* and b* parameters for both samples during storage, which was attributed to zeaxanthin release to the yogurt matrix, due to acidity changes. However, they also observed a decrease of L* values for both samples and control, indicating that the L* decrease observed was not caused by the incorporation of nanostructures, i.e., the incorporation of nanostructures did not affect the lightness of the yogurt [9].

The perception of the total color difference (Δ*E*) can be divided into several ranges: unnoticeable, Δ*E* < 1, only noticed by an experienced observer, 1 < Δ*E* < 2, noticed by an unexperienced observer, 2 < Δ*E* < 3.5 and clearly noticeable, 3.5 < Δ*E* < 5 [20]. This allows us to evaluate the quality and stability of the food product’s color properties. Both samples presented a slight increase of Δ*E* in the first 15 days, reaching a plateau until day 30 (see Figure 3d). Despite this, both samples presented statistically similar values of Δ*E* during the shelf-life and lower than one, showing that the differences were unnoticeable, and therefore both samples maintained their stability and quality regarding color during the shelf-life. Comunian et al. (2017) incorporated microcapsules with echium oil, phytosterol, and encapsulated sinapic acid and evaluated the color in terms of Δ*E* and color parameters (i.e., L*, a*, b*) for 30 days at 4 °C. The authors observed slight differences between the samples and between the days for the same sample; however, the Δ*E* was lower than one for all samples, showing that these color changes would not interfere with the consumers’ acceptance [12].

In conclusion, the incorporation of SLNs in yogurt conferred a strong yellow color but did not affect the stability and the quality of the yogurt, showing that curcumin-loaded SLNs can be used as natural dye as well as a delivery system.

### 3.5. Rheological Properties

#### 3.5.1. Flow Behavior and Apparent Viscosity

**Figure 5:**
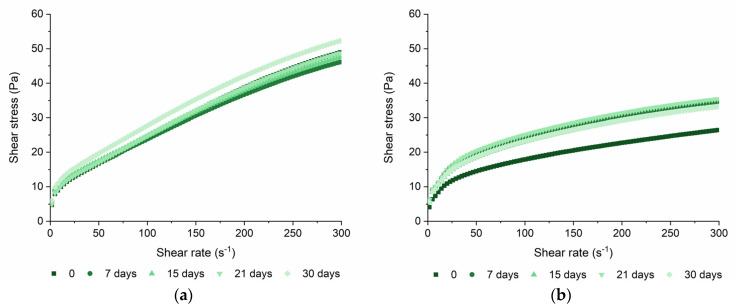
Flow curves of yogurt with SLNs (**a**) and control yogurt (i.e., plain yogurt) (**b**) during shelf-life.

The flow behavior and the viscoelastic profile are the main factors to understand the rheological behavior of foods under industrial processing, storage and oral processing conditions [21]. The relationship between shear stress and shear rate for the yogurt with SLNs and control yogurt during the shelf-life is presented in Figure 5a,b, respectively. The flow curves of both samples presented the existence of yield stress and shear-thinning behavior. This behavior is caused by the alignment of the biopolymer molecules with the shear force and the weak physical interactions between biopolymers, which means that their viscosity is dependent on the shear rate [22,23]. Similarly, other authors reported a shear-thinning behavior for the yogurts fortified with nanostructures and plain yogurts [24,25]. Once the presence of yield stress was observed in the curves, the flow behavior of both samples was characterized using the Herschel-Buckley model, Table 1. The Herschel-Buckley model fitted well with the experimental data with *R*^2^ > 0.99. The yield stress consists in the force/stress that needs to be applied to the material so that it starts to flow, which is linked to the existence of a structural network [23]. Yogurt with SLNs presented a range of yield stress values between 6.27 ± 0.44 Pa and 7.39 ± 0.39 Pa, while control yogurt showed a range between 1.69 ± 0.45 Pa and 0.70 ± 1.06 Pa for 0 and 30 days, respectively, Table 1. The yogurt with SLNs presented higher values of yield stress than the control yogurt (*p* < 0.05), showing that the incorporation of SLNs results in a stronger structure, probably due to the increase of dry matter by incorporating SLNs in powder form or the rearrangement of the gel matrix. Furthermore, the yield stress increased during the shelf-life (*p* < 0.05) for the yogurt with SLNs, while the control yogurt presented a higher value at the beginning but decreased in the first week and then remained close to zero. On the other hand, the yogurt with SLNs presented a lower consistency index (*K*) than the control yogurt during the shelf-life, despite presenting a slight increase of this parameter over time (see Table 1 (*p* < 0.05)). The flow behavior index (*n*) was lower than one for both samples, confirming the shear-thinning behavior of yogurts. These differences observed in the flow behavior parameters can be explained through the differences in composition, such as dry matter, fat content and the presence of emulsifiers. Despite the differences in the flow behavior parameters observed between the yogurt with SLNs and the control, the apparent viscosity was not significantly different between both samples and over the shelf-life, showing that the incorporation of SLNs did not impact the rheological stability of the yogurt during the shelf-life. On the contrary, other authors observed a decrease in the apparent viscosity with the incorporation of nanostructures due to the quantity of water added with the nanostructures [9,10], which did not happen in our work, probably due to the incorporation of SLNs in a powder form.

#### 3.5.2. Frequency Sweep

**Figure 6:**
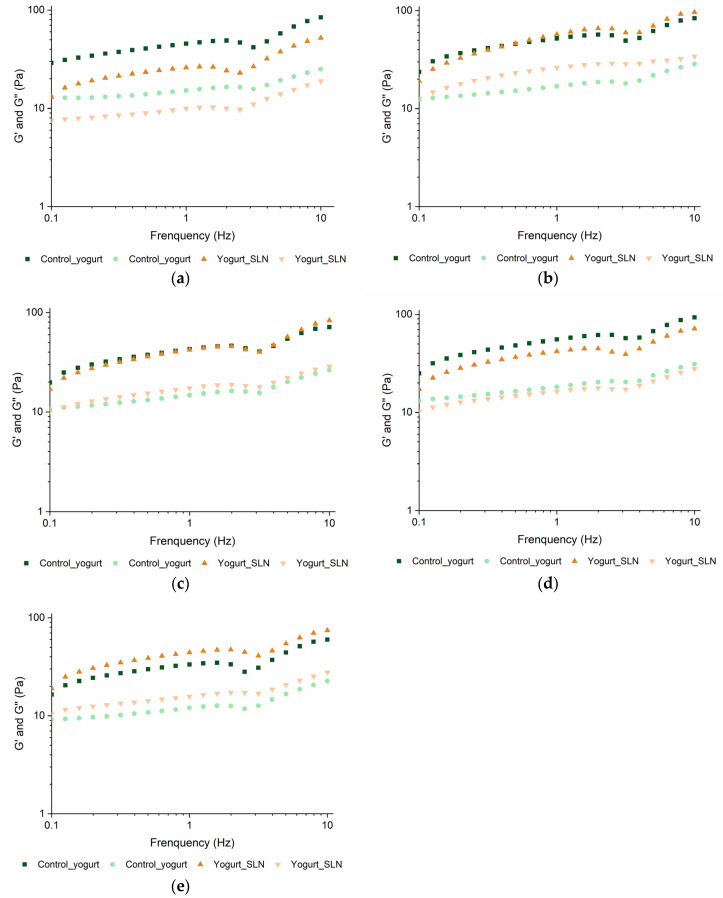
Frequency sweep of yogurt with SLNs and control yogurt (i.e., plain yogurt) at beginning (**a**), 7 days (**b**), 15 days (**c**), 21 days (**d**) and 30 days (**e**). Whereas 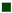 and 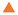 represent G′, 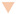 and ● represent G″.

The dependence of storage modulus (G′) and loss modulus (G″) on the frequency at different shelf-life times is presented in Figure 6. The yogurt with SLNs and the control present G′ values higher than G″ over the frequency range at all time points tested, showing a typical weak viscoelastic gel with an elastic structure. Furthermore, both samples present an increase of G′ and G″ with the increase of frequency, showing a typical physical gel behavior. These results are in agreement with the results obtained by other authors [18,22,25]. Overall, the yogurt with SLNs presented similar values of G′ and G″ to control yogurt during the shelf-life. Yet, the yogurt with SLNs presented a slight increase of the moduli over time, suggesting a strengthening of its structure, which corroborated the results from the flow behavior, where a small increase of yield stress was also observed over time. Despite this, the incorporation of SLNs in a powder form did not cause changes in the rheological behavior of the yogurt and maintained its stability during the shelf-life, corroborating the viscosity results.

## 4. Conclusions

The influence of the incorporation of SLNs in yogurt was assessed, evaluating yogurt’s physicochemical and rheological properties during its shelf-life (30 days). The yogurt with SLNs presented a 1.5% lower pH and an 11% lower titratable acidity than the control; however, both properties showed a similar tendency over time for fortified and control yogurts. Furthermore, the pH values obtained for yogurt with SLNs were within the consumer’s acceptance range and accepted for public safety. Regarding syneresis, SLNs incorporation promoted a 12% higher value than the control, which was maintained during the shelf-life. The incorporation of SLNs provided a strong yellow color to the yogurt and maintained the color stability of the yogurt, showing that curcumin-loaded SLNs can be used as a natural dye as well as a delivery system. Both the yogurt with SLNs and the control presented flow curves with yield stress and shear-thinning behavior. Despite the differences in the flow behavior parameters observed between the yogurt with SLNs and the control, the apparent viscosity was not significantly different between both samples and over the shelf-life. Furthermore, the incorporation of SLNs did not affect the viscoelastic behavior, showing a typical weak viscoelastic gel with an elastic structure, which was maintained during the shelf-life. Thus, the incorporation of SLNs did not impact the rheological stability of the yogurt during its shelf life.

Overall, the incorporation of curcumin-loaded SLNs did not affect the physicochemical and rheological stability of yogurt during the shelf-life (except for color, obviously). However, further studies should be carried out to evaluate curcumin’s bioaccessibility, sensory properties and the safety of yogurt with SLNs in order to increase the consumer’s acceptability of products where SLNs are used as carrier systems for nutraceutical compounds.

## Figures and Tables

**Table 1:** Rheological properties of yogurt with SLNs and control yogurt (i.e., plain yogurt) during the shelf-life: *σ_0_* (yield stress), *K* (consistency index) and *n* (flow behavior index) fitted with the Herschel-Bulkley model and *η_apparent_* (apparent viscosity) at a shear rate of 100 s^−1^.

Day	Yogurt	*σ*_0_ (Pa)	*K* (Pa.s^n^)	*N*	*η_apparent_* (Pa.s)
0	With SLNs	6.27 ± 0.44 ^A,a^	0.55 ± 0.05 ^A,a^	0.768 ± 0.011 ^A,a^	0.25 ± 0.01 ^A,a,b^
	Control	1.69 ± 0.45 ^B,a^	2.95 ± 0.55 ^B,a^	0.377 ± 0.020 ^B,a^	0.18 ± 0.02 ^B,a^
7	With SLNs	6.57 ± 0.80 ^A,a^	0.48 ±0.06 ^A,a^	0.778 ± 0.013 ^A,a^	0.23 ± 0.01 ^A,a^
	Control	0.00 ± 0.00 ^B,b^	5.80 ± 0.46 ^B,b,c^	0.316 ± 0.008 ^B,b^	0.25 ± 0.01 ^A,b,c^
15	With SLNs	6.62 ± 0.69 ^A,a^	0.51 ±0.05 ^A,a^	0.773 ± 0.011 ^A,a^	0.24 ± 0.01 ^A,a,b^
	Controlt	0.62 ± 0.72 ^B,a,b^	4.71± 0.57 ^B,b,d^	0.339 ± 0.013 ^B,b,c^	0.23 ± 0.01 ^A,b,c^
21	With SLNs	6.64 ± 0.50 ^A,a^	0.59 ± 0.06 ^A,a^	0.752 ± 0.017 ^A,a^	0.25 ± 0.01 ^A,a,b^
	Control	0.00 ± 0.00 ^B,b^	5.88 ± 0.76 ^B,c^	0.320 ± 0.011 ^B,b,c^	0.25 ± 0.02 ^A,b^
30	With SLNs	7.39 ± 0.39 ^A,a^	0.65 ± 0.05 ^A,a^	0.746 ± 0.012 ^A,a^	0.27 ± 0.01 ^A,b^
	Control	0.70 ± 1.06 ^B,a,b^	4.39 ± 1.44 ^B,d^	0.351 ± 0.040 ^B,b,c^	0.22 ± 0.03 ^B,c^

^a–c^ Different lower-case letters indicate a statistically significant difference over time for the same sample (*p* < 0.05). ^A,B^ Different uppercase letters indicate a statistically significant difference between samples for the same time (*p* < 0.05).

## Data Availability

The data presented in this study are available on request from the corresponding author.
